# Deep Lidar-Guided Image Deblurring

**DOI:** 10.3390/s25216786

**Published:** 2025-11-06

**Authors:** Ziyao Yi, Diego Valsesia, Tiziano Bianchi, Enrico Magli

**Affiliations:** Department of Electronics and Telecommunications, Politecnico di Torino, 10129 Torino, Italy; ziyao.yi@polito.it (Z.Y.); tiziano.bianchi@polito.it (T.B.); enrico.magli@polito.it (E.M.)

**Keywords:** image deblurring, lidar depth map, deep neural network

## Abstract

The rise in portable Lidar instruments enables new opportunities for depth-assisted image processing. In this paper, we study whether the depth information provided by mobile Lidar sensors present in recent smartphones is useful for the task of image deblurring and how to integrate it with a general approach that transforms any state-of-the-art neural deblurring model into a depth-aware one. To achieve this, we developed a continual learning strategy integrating adapters into U-shaped encoder–decoder models that efficiently preprocess depth information to modulate image features with depth features. We conducted experiments on datasets with real-world depth data captured by a smartphone Lidar. The results show that our method consistently improves performance across multiple state-of-the-art deblurring baselines. Our approach achieves PSNR gains of up to 2.1 dB with a modest increase in the number of parameters, which demonstrates that utilizing true depth information can significantly boost the effectiveness of deblurring algorithms with the encoder–decoder architecture.

## 1. Introduction

Image deblurring stands as a crucial task in the low-level vision realm, especially in the digital age where cameras have become ubiquitous across a wide array of personal electronic devices such as smartphones and tablets. These devices can easily capture blurred images due to various factors, including shaky hands due to limited space and budget for anti-shake hardware, as well as poor focusing.

Deblurring algorithms have been studied for decades with the aim of recovering clear and sharp images from those with indistinct or blurred details. Mathematically, deblurring is an ill-posed inverse problem, which requires strong priors about the nature of the images to be recovered in order to be effectively regularized. Indeed, with the recent successes of data-driven methods based on neural networks, learning-based deblurring algorithms also have evolved rapidly, from convolutional and Recurrent Neural Networks [1,2] to Transformers [3,4,5] and from Generative Adversarial Networks [6] to diffusion models [7]. The success of all of these can be largely attributed to their ability to learn sophisticated image features from training data. However, comparatively fewer works [8,9,10] have focused on ways to incorporate side information, mainly in the form of event cameras, segmentation information, or optical flow, as an alternative way to help with the regularization of the deblurring inverse problem. Historically, guided filters [11] have been used to modulate the filtering process with a guidance signal for this purpose.

Regarding this, multimodal imaging platforms, which combine multiple kinds of imaging devices, are currently gaining popularity. In particular, recent mobile devices, such as the Apple iPhone and iPad [12], are now being equipped with Lidar sensors to provide depth information that can be used for 3D scanning capability. Such Lidars are time-of-flight sensors which send a grid of light pulses and measure the return time to estimate distance at multiple points, thus providing a depth map of the scene. Active sensing instruments are particularly interesting, as they can complement passive optical cameras. In particular, these can be prone to generating blurry images in situations requiring, even slightly, longer exposures, integrating received light over a longer period of time but being sensitive to camera shakes on handheld devices. Alternatively, errors in focusing under challenging conditions may also result in blurred images. This raises the question of whether true depth information from Lidar sensors, particularly smartphone ones, can be effectively used to regularize the deblurring problem and improve image quality.

Several challenges need to be overcome in order to answer this question. First, our focus will be on smartphone Lidars and cameras, as this is, possibly, the most widespread multimodal sensing platform at the moment. However, mobile Lidars have significant limitations in spatial resolution due to their size and cost, so it is not obvious whether they can provide sufficient information. Moreover, state-of-the-art image restoration models based on neural networks require large datasets to be effectively trained. At the moment, there is no existing dataset of blurry images with associated Lidar depth maps captured by smartphones, and assembling one of large size to enable effective training from scratch is indeed a challenging task.

In this work, we aim to answer the question of whether smartphone Lidar can boost image deblurring performance. We propose a novel approach that integrates depth maps with blurred RGB images in a way that is able to address the aforementioned challenges. In particular, we propose a continual learning approach where conventional encoder–decoder deblurring models are augmented and finetuned with adapters to incorporate depth information. We design a novel adapter neural network inspired by the classic guided filter to process depth maps and use their features to modulate the features extracted by any state-of-the-art image restoration model. The adapter also deals with the limited resolution of mobile Lidar depth maps by including a super-resolution operation that is capable of preserving their piecewise constant nature when upscaling them to the target resolution.

In summary, our main contributions can be regarded as follows:

- We propose a novel approach to image deblurring that augments state-of-the-art models with depth information from smartphone Lidar sensors.
- We propose a novel continual learning strategy that finetunes state-of-the-art deblurring models with adapters that process depth information and use it to module the main model features.
- We propose a design of the adapter architecture that is inspired by the classic guided filter to effectively use depth to modulate image features.
- We show that the true depth information obtained by mobile Lidar sensors improves image deblurring performance, as experimentally verified with real-world Lidar data.

This paper is organized as follows: Section 2 reviews the relevant background and related work on image deblurring and continual learning. Section 3 presents the proposed framework in detail, including depth super-resolution, depth adapters, and continual learning strategies. Section 4 reports the experimental results, including quantitative comparisons, qualitative analysis, and ablation studies. Finally, Section 6 concludes this paper and outlines potential directions for future work.

## 2. Background and Related Work

### 2.1. Image Deblurring

Deblurring is a classic ill-posed inverse problem that has a long history of methods being used to attempt to reconstruct a sharp image from blurred observations. Mathematically, the observed image y is modeled as the convolution between a blur kernel k and the original image x:

(1) $$\begin{array}{c} y=k⊛x, \end{array}$$

where ⊛ is the convolution operator. The knowledge of the blur kernel discriminates between non-blind and blind deblurring problems.

Traditional approaches cast reconstruction as the solution of a regularized least squares optimization problem, where the regularizer incorporates prior knowledge about the original images. The extensive literature has focused on devising sophisticated image priors [13,14,15,16,17,18].

Deep learning approaches have enjoyed great success thanks to their ability to learn the prior directly from training data [19,20,21]. A common approach is supervised learning, where pairs of blurred and unblurred pictures are available for training [1,3,4,6,22,23,24,25,26]. Zhang et al. [1] proposed a network composed of three CNNs and an RNN to be used as a deconvolution operator. DeblurGAN [6] and DeblurGAN-v2 [22] introduced adversarial learning for deblurring tasks. MRLPFNet [23] and MSSNet [24] used multiscale architectures to jointly explore image details and main structures for deblurring. Given the success of Transformers in both language processing [27] and vision [28], they have also gained attention in the deblurring literature. For example, IPT [3] first applied standard Transformer blocks and trained on large-scale datasets. More recently, Uformer [25] designed a general U-shaped Transformer-based structure, which proved to be efficient and effective for image restoration. Stripformer [5] constructed intra- and inter-strip tokens to reweight image features. Restormer [4] proposed a Transformer-based architecture that is able to learn long-range dependencies while remaining computationally efficient. On the other hand, NAFNet [26] proved that simple neural network designs using gating mechanisms can obtain excellent performance without the need for the expensive self-attention operation of Transformers.

Some works have studied the use of information from other domains such as segmentation maps [8,10], optical flow [9], and event camera data [29,30] to better regularize the reconstruction process and improve image quality. For example, EDMF [30] leverages event camera data through spatio-temporal voxel representation and multimodal fusion to restore high-frequency details in motion-blurred scenes. Nury et al. [31] integrate semantic features from CLIP and Stable Diffusion into a two-stage diffusion-based framework, enhancing perceptual and structural restoration. A small number of works have also explored using depth information to improve deblurring performance. The authors of Ref. [32] proposed a hierarchical depth estimation based on region trees to progressively generate credible blur kernel estimates. Li et al. [33] first extracted a depth map and adopted a depth refinement network to restore the edges and structure in the depth map. Li [34] introduced a two-stage framework combining multimodal-guided diffusion with depth-aware fusion to improve restoration consistency across defocus regions by estimating depth with DepthAnything [35]. Notably, all these works estimated depth information from the blurry image itself. This is in contrast with our work, which uses external information in the form of Lidar data, posing the issue of ensuring its quality and proper fusion but, theoretically, providing truly independent measurements.

Deep learning approaches to image deblurring require careful dataset selection, in order to ensure the availability of sufficient data for training that are representative of the problem of interest. Some datasets for deblurring tasks are generated by averaging frames from high-frame-rate videos in order to simulate blurs due to long exposures. For example, the GoPro [36] dataset contains 240 fps videos taken with GoPro cameras, and it then averaged a variable number (7–13) of successive frames to produce blurs of different strengths. Similarly, the REDS [37] and DVDs [38] datasets were constructed for video deblurring. Besides averaging video frames, Levin [39] collected real blurred images by capturing images on the wall while shaking cameras and providing blur kernels. The authors of [40,41,42] also provided images affected by real blur.

In this work, we are interested in studying the performance of deblurring when in the presence of depth data acquired by mobile Lidars. No such dataset is currently readily available for this task. However, the ArkitScenes [43] dataset presents a large number of indoor scenes acquired with Apple mobile devices, iPhones and iPads, together with registered Lidar scans. In particular, depth information is provided by both Lidar scans from the mobile sensors and scans from a high-end professional Lidar (Faro Focus S70). While RGB images are not affected by blur, it is possible to simulate it using standardized blur kernels [39] used for benchmarking deblurring methods. This allows us to study the effectiveness of depth information in regularizing the deblurring procedure. The availability of Lidar data with both low and high spatial resolution also allows to study its impact on the restoration process.

### 2.2. Continual Learning

The effectiveness of deep learning models may increase significantly, as they are scaled in size and the amount of training data. Many recent models can be very large and expensive to train. It is thus desirable to incorporate new requirements in the least expensive way possible when one wants to leverage them for a task that is not training [44,45,46]. This has brought about recent interest in novel ways to continue training such as low-rank adapters (LoRA) [47] and HyperNetworks [48]. This field is generally known as “continual learning”, incremental learning, or lifelong learning, and multiple strategies have been developed to accomplish its goals, e.g., regularization, model expansion, and rehearsal [49]. In the case of this paper, which involves extending existing models to support a new modality, the scheme we develop falls in the general class of model expansion, which adds parameters to learn new tasks or classes or incorporate new modalities.

An example of continual learning methods in computer vision is represented by VPT [50], which introduces less than 1% of new trainable parameters in the input space while keeping the model backbone frozen to solve various downstream image recognition tasks. Gao et al. propose CPrompt [51] for more aligned training and testing, which surpasses its prompt-based counterparts and achieves state-of-the-art performance on multiple continual learning benchmarks. Besides pure vision prompt learning, multimodal prompt learning has also been proposed. MaPLe [52] improves alignment between the vision and language representations. Zhu et al. [53] developed Visual Prompt multimodal Tracking (ViPT), which outperforms the full finetuning paradigm on multiple downstream tracking tasks including RGB+Depth, RGB+Thermal, and RGB+Event tracking.

In the image restoration literature, Potlapall et al. [54] proposed PromptIR, which uses prompts to encode degradation-specific information and then guides the restoration network dynamically. ProRes [55] and PromptGIP [56] add additional images as prompts. MiOIR [57] adopts sequential prompt learning strategies to adapt to multiple restoration tasks. However, these methods are focused on the same image domain or focus on the solution of new restoration tasks, neglecting to explore the possibility of using information from other domains. In our work, we need to carefully devise continual learning strategies to modulate existing image features at the pixel level in order to incorporate information from a different modality with different properties such as radiometry and spatial resolution.

## 3. Materials and Methods

This section presents the proposed approach to studying the effectiveness of mobile Lidar depth maps, as well as novel solutions to effectively incorporate this information into state-of-the-art deblurring models.

### 3.1. Intuition

Image blur in photos is usually the result of improper focusing or motion due to long exposure times, e.g., because of hand shaking, and results in object boundaries or edges that appear indistinct and smeared. Lidar is an active instrument that is capable of measuring the distance of surfaces in the scene from the camera. Being active, it does not suffer from the same limitations as RGB cameras. For instance, in a low-light setting, the need to integrate light for a sufficiently long time results in blur due to even modest shaking. However, Lidar measurement is unaffected by this and can discern sharp object boundaries, provided a sufficiently high-resolution capture, in the form of a depth map. Since the results in the literature [33] showed that even estimating depth from blurry images alone can be useful, the conjecture we seek to verify is whether a real depth map, even of modest resolution, provided by an independent instrument can effectively guide the restoration process towards sharper results, particularly around objects, leading to a more accurate and visually pleasing deblurred image.

Formally, let us call y the blurry observed RGB image and d the depth map acquired by the Lidar instrument, possibly at a lower spatial resolution. We seek to develop a joint multimodal model $f_{\theta }\left(y,d\right)$ that can estimate the clean image x. In developing this model, we need to consider two main factors: (i) joint training data with blurry images and associated Lidar depth maps are scarce and will be, in general, difficult to acquire on large scales; (ii) future advancements in the state of the art will generally focus on unimodal image deblurring. This leads us to formulate the joint model as a factorized one, i.e.,

(2) $$\begin{array}{c} f_{\theta }\left(y,d\right)=g_{\theta_{g}}\left(y\right)\circ h_{\theta_{h}}\left(d\right), \end{array}$$

where a unimodal deblurring model from the state of the art $g_{\theta_{g}}\left(y\right)$ can be combined with a depth processor $h_{\theta_{h}}\left(d\right)$ which takes care of extracting suitable features from the depth data and combining them with those from the deblurring model, possibly in a manner that is as universal as possible. A continual learning strategy is desirable to train such a joint model and exploit the rich datasets used to train unimodal deblurring models. With this scheme, the limited multimodal training data can be used to train only the $\theta_{h}$ parameters and apply a small update to the pretrained $\theta_{g}$ parameters.

With this in mind, the following section will focus on the main aspects that are critical for the overall performance of the multimodal model and its wide applicability, namely the need to extract high-quality depth features, also addressing the limitations in the spatial resolution of the Lidar capture and ensuring the fusion operator ∘ is well-constructed and effective at merging the depth information.

### 3.2. Lidar-Guided Deblurring

**Figure 1:**
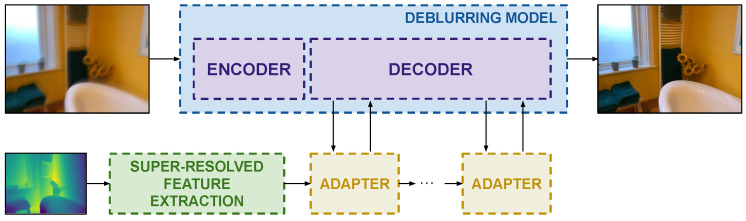
The pipeline of a generic depth-guided image deblurring model. Depth guidance is super-resolved, and features are extracted and injected by efficient adapters at the decoder side.

**Figure 2:**
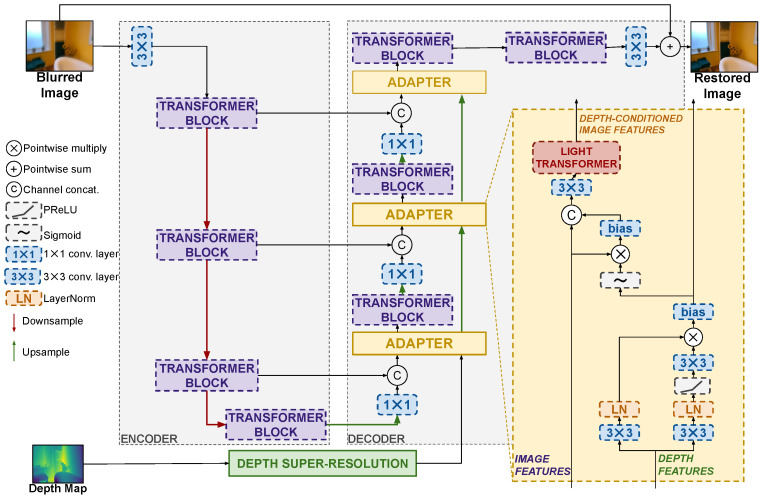
The Depth–Restormer architecture example. The adapter is added on each level of the decoder part, which is in front of the transformer block. The activation function used in the adapters is PReLU. For the convolutional layers, the details are as follows: The 3×3 convolution uses kernel size = 3, stride = 1, and padding = 1. The 1×1 convolution uses kernel size = 1, stride = 1, and padding = 0.

This section presents the previously mentioned key ingredients for designing general Lidar-guided image deblurring models. A high-level overview of them is presented in Figure 1, and a specific adaptation to the Restormer architecture [4] is shown in Figure 2.

#### 3.2.1. Quality of Depth Features

Depth maps are approximately piecewise smooth images, which, ideally, should show sharp transitions between objects as they appear at different relative distances or are separated by a background. However, the quality of real depth maps, as captured by Lidar instruments, can be variable depending on a number of factors. In particular, they might present missing values due to lost light return when a surface scatters the transmitted pulse away from the camera. This can be coarsely fixed by inpainting based on neighboring measurements or with some RGB guidance. We remark that in this paper, we deal with smartphone Lidars and are limited by what available datasets offer in terms of the analysis of robustness to such phenomena by only having access to partially inpainted depth maps. This is, in general, suboptimal as access to raw data could provide better information, but we will show that this is nevertheless already effective for the deblurring task. Additionally, the spatial resolution of the depth map can be limited due to manufacturing size and cost constraints, particularly on mobile devices. For example, the Apple iPad Pro used in the ARKitScenes dataset [43] captures depth at a resolution of 256×192 pixels, which is substantially smaller than the resolution of the RGB camera.

**Figure 3:**
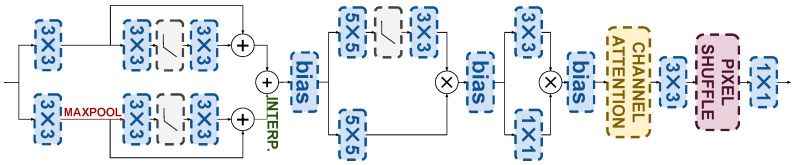
Depth super-resolution architecture.

It is thus clear that feature extraction must be combined with a super-resolution model to extract depth features that are as useful as possible to guide the deblurring model. Indeed, Section 4.4.2 will show that the naive upsampling of the depth map provides significantly diminished guidance, leading to more modest deblurring improvements. Depth map super-resolution is known to require ad hoc models [58,59] to preserve the piecewise smooth nature of depth images and their sharp edges. In this work, we propose a lightweight design of a depth super-resolution network whose features serve as the starting point of the depth processing model $h_{\theta_{h}}$. The architecture we use is detailed in Figure 3. The Depth-SR network adopts a two-branch architecture designed for parallel execution to maintain computational efficiency. Depth features at multiple scales are extracted using a pyramid structure with max-pooling and skip connections. Then the features are processed through two parallel branches of linear transformation layers, with one branch incorporating a non-linear activation function to enhance representational capacity. The outputs of the two branches are fused via a gating function, followed by a lightweight channel attention module [26] to further enhance the depth features. A supervised pretraining process is used by exploiting the ARKitScenes data, which provide paired captures from the low-resolution mobile Lidar of the iPad Pro and the high-resolution Faro Focus S70 Lidar. Notice that the input for the super-resolution process is just the depth map, and the RGB image is not used to prevent contamination with blurry data. After this pretraining, the last layer, which projects features back to image space, is removed to directly provide deep features to the adapter modules described in the next section. Indeed, this design choice is motivated by recent results showing that the conventional approach to integrating depth maps [60] in other models via concatenation or cross-attention with the depth map itself can be suboptimal [61]. This is because the depth map can be regarded as a shallow feature, and its combination with deeper features may result in information misalignment. Since we seek to integrate depth features with deep features at the decoder stage of encoder–decoder restoration models, it is thus important to leverage deep depth features.

#### 3.2.2. Continual Learning via Depth Adapters

The fusion of multimodal information in deep neural networks can be performed in several different ways, depending on design constraints and the type of information. For example, popular basic methods use early fusion by concatenating the raw inputs or shallow features of the different modalities. When a pretrained unimodal model is available, this is a suboptimal solution, as the new joint model will not fully exploit the new modality.

In our problem, the side information provided by the depth map can be regarded as guidance regarding the edges to be used to regularize the main features extracted from the image. The image filtering literature before deep learning has shown that the use of the guided filter [11] is a compelling solution to this problem. The guided filter can be regarded as a spatially variant filter whose coefficients are derived from the second-order statistics of the guidance signal. Referring to Equation (11) in [11], the filtered image q at pixel *i* is obtained as a function of guidance (depth in our case) d and the image to be filtered p by the following:

(3) $$\begin{array}{cc} q_{i} & =\sum_{j}W_{ij}\left(d\right)\cdot p_{j} \end{array}$$

(4) $$\begin{array}{cc} W_{ij} & =\frac{1}{{\left|\omega \right|}^{2}}\sum_{k:\left(i,j\right)\in \omega_{k}}\left(1+\frac{\left(d_{i}-\mu_{k}\right)\left(d_{j}-\mu_{k}\right)}{\sigma_{k}^{2}}\right) \end{array}$$

where $\mu_{k}$ and $\sigma_{k}^{2}$ are the mean and variance of a local window $\omega_{k}$.

We propose a generalization of the guided filtering scheme in the form of lightweight neural network adapters. These adapters process a multiresolution sequence of depth and image features and, similarly to the guided filter, modulate the image features using the depth ones. The design of our adapter architecture draws inspiration from Equation (4) to devise a suitable sequence of neural operations, albeit we do not seek an exact mathematical mapping of the guided filter equations. More specifically, the proposed adapter architecture is shown in Figure 2 for an encoder–decoder restoration model such as Restormer. Referring to the figure, the adapter architecture can be broken down into a sequence of three main operation, each justified by the analogy with the guided filter and similar approaches to adapter designs in the literature [54]: (i) a convolutional attention operation deriving second-order depth features; (ii) the modulation of the image features by means of the depth features; and (iii) the adaptive merging of modulated features into the main network via a lightweight Transformer.

The input depth features are first processed by a convolutional attention operation, which mimics the second-order statistics of the original guided filter. Referring to Equation (4), we can see how the image to be filtered is modulated by weights obtained as the second-order statistics of guidance d (product of $d_{i}$ and $d_{j}$), normalized by the local mean and variance. The convolutional attention operation mimics this by means of two parallel branches processing the input depth features with a convolutional layers and LayerNorm normalization, which are then multiplied together in an attention-like manner which allows us to recall the second-order nature of the features. The result is then passed through a sigmoid to stabilize the operations and then modulates the image features by means of multiplication with them, as in Equation (3). The analogy with the guided filter stops here, but the adapter has a final operation where the modulated features are concatenated to the original image features for further processing by a convolutional layer and a lightweight Transformer before being reintroduced into the deblurring model. The existence of this operation is justified by its ability to let the model adaptively decide whether to reject the modulated image features or not, which might be useful in the case of unreliable guidance. Similar designs have been shown to be effective in adapters for other tasks [54]. The lightweight transformer from [4] is composed of a sequence of multi-Dconv head transposed attention (MDTA) and a Gated-Dconv feed-forward network (GDFN). The MDTA block projects the fused feature to query (Q), key (K), and value (V) by utilizing only a single attention head to minimize computational complexity, and the GDFN regulates information flow across hierarchical levels by employing a gating mechanism combined with pixel-wise convolutions, enabling each level to concentrate on fine-grained details that complement features captured at other levels.

We propose to incorporate adapters only at the decoder side of encoder–decoder deblurring models. This follows other approaches in the literature for similar problems [54], where no substantial benefit was observed in including adapters both at the encoder and decoder. This can be explained by the fact that the decoder is concerned with reconstructing spatial details, and hence it is where the guidance signal is the most useful, rather than the derivation of abstract features at the encoder side. The experimental results also validate this hypothesis. We also remark that the depth features processed by the convolutional attention operation of the adapter are also propagated forward to the next stage and possibly upsampled.

Introducing the previously described adapters into any state-of-the-art encoder–decoder model allows us to use a continual learning strategy where deblurring models pretrained on data where depth information was not available can be finetuned together with the training of the adapters in a supervised way to obtain a depth-guided model. We propose to fully finetune the pretrained model and the adapters for the best results.

#### 3.2.3. Example: Depth–Restormer

The proposed approach based on depth super-resolution, adapters, and continual learning is well-suited to introduce Lidar depth information into any state-of-the-art image deblurring model. As an example, we report the full architecture of the well-known Restormer model [4] with the proposed additions to deal with Lidar depth, resulting in the Depth–Restormer model shown in Figure 2. In the Restormer architecture, the encoder progressively reduces the image resolution after every Transformer block with skip connections to the corresponding layers in the decoder half. Notice how we introduce the adapters in the decoder stage, after every upsampling operation. Similar considerations can be made for any state-of-the-art deblurring method with an encoder–decoder structure or with an inherent symmetric design between the first and second halves of the model, which is most of the existing approaches. Indeed, Section 4 reports on experiments on the depth-enhanced versions of multiple state-of-the-art models, including Restormer [4], NAFNet [26], Stripformer [5], and DeblurDiNATL [62].

## 4. Experimental Results

This section reports the experimental results to validate several points of interest. First and foremost, we seek to answer the question of whether image quality is improved by providing mobile Lidar depth maps. This is conducted by presenting the results on several state-of-the-art deblurring architectures adapted following the proposed approach. Next, we validate the design of the proposed approach, particularly regarding the need for depth super-resolution, and the adapter design.

### 4.1. Datasets

In our experiments, we use a subset of the ArkitScenes dataset [43], specifically the portion used for RGB-D-guided upsampling, which contains 29,264 image depth pairs in the training set. For validation, we randomly sample 500 pairs from the original validation set. Image blur is simulated by randomly choosing a blur kernel from a set of standard benchmark kernels, following the approach from [39]. The blur kernel sizes defined in [39] range from 11×11 to 19×19 in the original implementation. These kernels were designed for rescaled scenes of size 256×256 pixels. Since our input images are significantly bigger at 1440×1920 pixels, we rescale the kernels by a factor of 1440/256≈5.6 in order to preserve the relative ratio between the blur diameter and the image dimensions. This adjustment ensures that the simulated blur maintains similar strength in terms of spatial frequency attenuation characteristics as in the original setting, resulting in more realistic and perceptually consistent blur patterns across the different resolutions. Meanwhile, a novel dataset of real blurred images with associated mobile depth maps (LICAM dataset [63]) is used as an additional evaluation, which contains 200 training images of size 1024×1024 and 180 test images of the same size.

The depth super-resolution network is pretrained on the same ArkitScenes dataset by using the low-resolution depth maps from the iPad Lidar as input and the high-resolution depth maps from the Faro Focus S70 Lidar as ground truth. The ground truth data contain some pixels with invalid measurements which are masked to be discarded in the loss computation.

### 4.2. Implementation Details

We selected four main state-of-the-art deblurring models to be tested with and without Lidar augmentation: Restormer [41], NAFNet [26], Stripformer [5], and DeblurDiNATL [62]. The versions with the proposed Lidar depth improvements using adapters and the continual learning strategy are denoted with the prefix “Depth-*”. Hyperparameters for our experiments are shown in Table 1. In particular, this table reports the number of feature channels in the depth adapters and the SR network. Training generally follows the protocols outlined in the original papers in terms of image patch sizes, with 128×128 patches for Restormer and Stripformer and 256×256 for NAFNet and DeblurDiNATL. In the training process, first, the SR network is pretrained on the ARKitScenes data using L1 loss:

(5) $$\begin{array}{c} L_{SR}={∥\hat{d}-d∥}_{1} \end{array}$$

where $\hat{d}$ is the super-resolved depth map estimated by the network and d the high-resolution ground truth from the Faro S70 Lidar. For this pretraining, the Adam optimizer is used with $\beta_{1}=0.9$ and $\beta_{2}=0.999$ and a fixed learning rate equal to $10^{-4}$ for 50 epochs.

The backbone model weights are fully finetuned from the pretrained values on the GoPro dataset, as provided from the original implementation of the backbones, and the adapters are trained with a loss that is a combination of L1 and cosine distance:

(6) $$\begin{array}{c} L=L_{1}+L_{CD}={∥\hat{x}-x∥}_{1}+\left(1-\frac{{\hat{x}}^{T}x}{∥x∥∥\hat{x}∥}\right) \end{array}$$

where $\hat{x}$ is the deblurred image and x the ground truth image. The initial learning rate is $5\times 10^{-5}$ and gradually decays to $1\times 10^{-7}$ every 50 epochs with the cosine annealing decay policy, and the Adam optimizer has $\beta_{1}=0.9$ and $\beta_{2}=0.999$. The published version of the method and the depth-enhanced one are trained on the same data and with the same protocol to ensure a fair comparison.

For the experiment on the LICAM dataset, the entire model is finetuned from the weights trained on the ARKitScenes data using LPIPS loss for 25 epochs using an Adam optimizer with $\beta_{1}=0.9$ and $\beta_{2}=0.999$ and with a fixed learning rate equal to $10^{-4}$. This choice is motivated by slight misalignments between the ground truth and blurred images for this dataset, which make the L1 or cosine distances unreliable.

The deblurring results on the ARKitScenes dataset are evaluated in terms of the widely used Peak Signal-to-Noise Ratio (PSNR), Structural Similarity Index Measure (SSIM) metrics, and the LPIPS distance [64] for a more perception-oriented metric. For the LICAM dataset, only the LPIPS distance is used to avoid the aforementioned issues.

Experiments were performed on four Nvidia A6000 GPUs. Depending on the specific neural network model, training requires approximately 2–3 days on the ArkitScenes dataset and two hours on the LICAM dataset. The code is available at https://github.com/diegovalsesia/lidardeblurring (accessed on 30 October 2025).

### 4.3. Main Results

**Figure 4:**
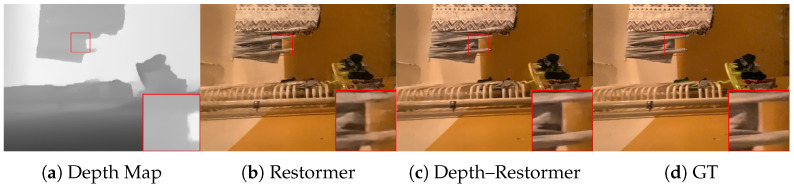
Left to right: Mobile Lidar depth map, Restormer deblurred image, Depth–Restormer, ground truth. As shown in detail, Depth–Restormer has sharper object edges.

**Figure 5:**
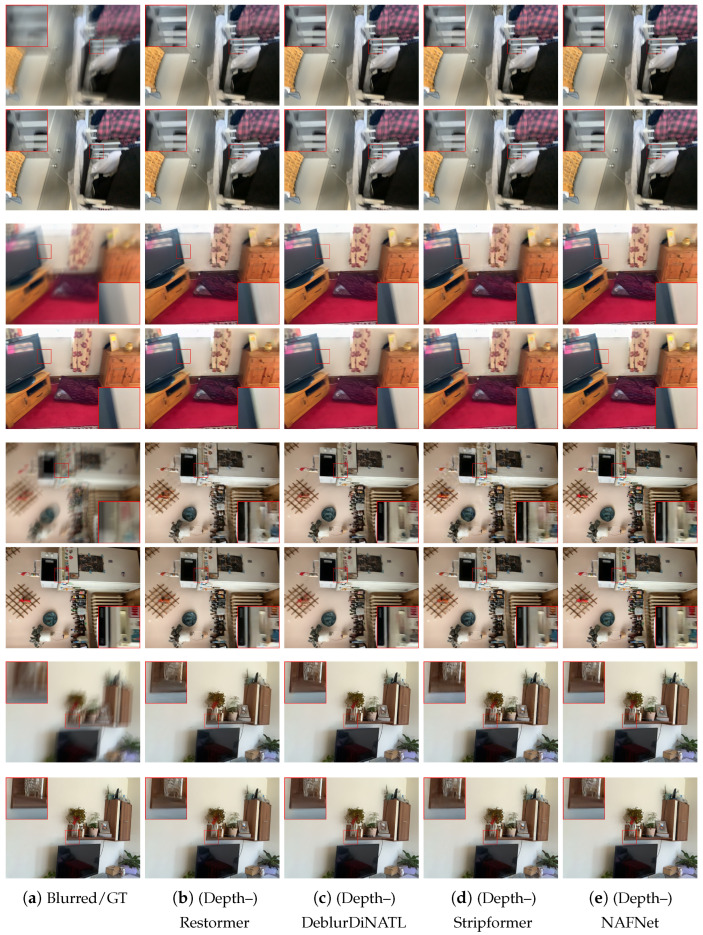
A visual comparison of the deblurring results for the considered state-of-the-art models. For each scene, the top row presents the results of the original model, while the bottom row presents the results of the depth-enhanced model.

We first assess whether mobile Lidar data can improve the deblurring results on the selected state-of-the-art architectures. The results are shown in Table 2. We can notice that the use of Lidar data generally provides a significant improvement in deblurring performance. The only exception is the NAFNet architecture where we still observe improvement but more modest ones. This could be explained by both the unusual network design of NAFNet and the fact that a saturation point was reached in the ability to deblur with any model (indeed, NAFNet achieves a baseline quality significantly better than that of the other models). We can also observe that the increase in the number of parameters is modest with respect to the size of the original models. The runtime results report the inference latency as measured on an Nvidia A6000 GPU and show that the integration of depth via adapters only marginally increases complexity. Further work is need for low-complexity models that could be directly run on smartphone devices. The qualitative results are reported in Figure 4 and Figure 5. In this figure, for each scene, the top line is the result of the four state-of-the-art conventional deblurring models without depth information, while the bottom line shows the results of the depth-enhanced models. It can be noticed that in correspondence to object boundaries, the depth-enhanced models significantly reduce ghosting effects. Figure 4 reports the same result for the Restormer architecture while showing depth information.

Additionally, we present an experiment on a recently introduced dataset of low-light smartphone images affected by motion blur and noise, with registered Lidar depth maps and ground truth images [63] of size 1024×1024. For this experiment, we finetune models pretrained on ARKitScenes using the LPIPS perceptual loss for 25 epochs with a learning rate of $10^{-4}$ on the images in the training split. Evaluation on the test split also uses the LPIPS distance for perceptual distortion as it is more robust to slight misalignments with respect to the ground truth data. The results are reported in Table 3 and confirm the effectiveness of depth information.

Finally, we present an experiment aimed at evaluating the quality of the deblurred images by means of performance on a downstream semantic segmentation task. Since the available datasets do not have ground truth segmentation maps, we devise a procedure where a pretrained Segment Anything Model (SAM) [65] is used on the sharp ground truth images of the ARKitScenes test set to generate ground truth segmentation maps. Then, the SAM is used to estimate segmentation maps from the deblurred images with and without depth guidance using the Restormer architecture. We report a 99.37% accuracy for the model without Lidar depth information and 99.68% for the model with depth information. Notice that the accuracy values are quite high due to blur only marginally affecting the semantic segmentation. Nonetheless, a measurable improvement on the downstream task confirms the effectiveness of depth information.

Overall, these results demonstrate that mobile Lidar depth maps, despite their relatively low resolution, can successfully regularize the deblurring process when properly used with the proposed scheme.

### 4.4. Ablation Study

In the ablation study, we carefully analyze our design decisions to validate their effectiveness. This study concerns the quality of the depth maps and the continual learning strategy used to fuse them with the deblurring model. All the ablation results use the Restormer architecture as the baseline.

#### 4.4.1. Impact of Real Lidar Depth Maps

**Figure 6:**
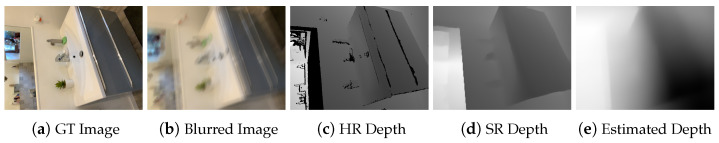
Comparison of depth maps. From left to right: ground truth image, blurred image, high-resolution depth map from Faro Focus S70 Lidar, super-resolved depth map from iPad Pro, depth map estimated from blurred image by [35].

Some approaches in the literature [32,33] attempt to use estimated depth maps to aid image deblurring, while [66] utilized both real and estimated depth maps, achieving good performance. However, the literature generally lacks comparisons between the use of real and estimated depth maps, and mobile Lidars have not yet been considered for image restoration problems. We argue that depth map estimation from a blurry image can only provide additional image features that might have some use in the reconstruction process but do not really provide additional side information, as an independent Lidar would. Therefore, in this study, we compared the PSNR of the deblurred image obtained when real Lidar depth maps are used and when, instead, a depth map is estimated from the blurry image. The state-of-the-art Depth Anything [35] model is used to estimate the depth maps. Because the model generates depth maps with the same resolution as the blurred images, the depth map super-resolution block is not used. From Figure 6, we can see that the depth map generated from the blurred image lacks the explicit geometric information, particularly regarding object edges, that is present both in the high-resolution depth map from the high-end Lidar and the super-resolved depth map of the mobile Lidar. The quantitative results in Table 4 confirm the results of the previous literature [32,33] in that even estimating the depth map from blurry images provides some degree of regularization to the deblurring process, leading to some improvements. However, Lidar depth maps provide a more significant improvement in performance, proving that the independent side information captured by the Lidar instrument, even if at modest resolution, can boost image quality.

#### 4.4.2. Impact of Lidar Depth Super-Resolution

**Figure 7:**
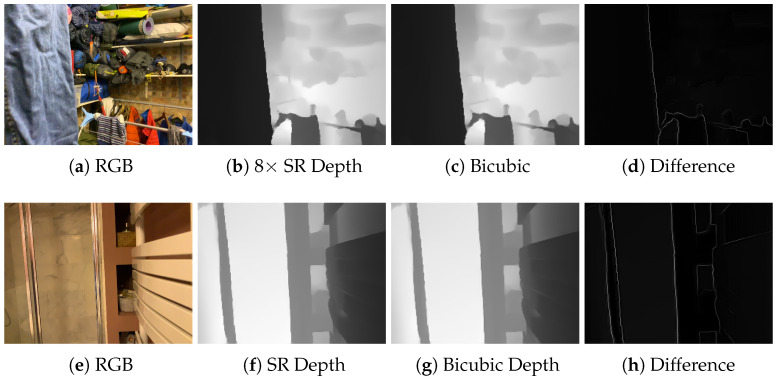
The neural network super-resolved iPad depth map better preserves sharp edges after the upscaling operation, leading to improved deblurring performance.

While we observed that a super-resolved mobile Lidar depth map can increase deblurring quality more than estimating it from the blurred image, we still need to analyze the sensitivity of the process to the resolution of the depth map. Therefore, we conducted an experiment with different depth map super-resolution scales, specifically four times and eight times, and with different methods, namely bicubic interpolation, and we also compared the results with high-resolution depth maps provided by the Faro Focus S70 Lidar. The results are shown in Table 5. We first notice that the neural network approach to depth super-resolution significantly outperforms bicubic interpolation. Bicubic interpolation is mainly limited by being a general interpolation method fitting cubic polynomials, and as such, it does not exploit priors specific to depth data such as the fact that they are approximately piecewise constant. However, we notice that the proposed approach with bicubically interpolated depth maps is still improved over that not using depth maps. We also confirmed this result by running the experiment on the Stripformer architecture, where using bicubic upsampling achieves a PSNR equal to 35.89 dB instead of the 35.17 dB achieved by the model without depth, although this is still lower than the 36.34 dB achieved with the SR neural network. These results suggest that, despite its limitations, bicubic interpolation could be used as an effective baseline in the absence of a dedicated SR network. As an example, this could happen for new Lidar sensors that might be significantly different from the current iPhone ones and for which paired training data with HR Lidar depth maps might not be available. We also notice that the 8× super-resolution factor, which matches the ratio between the RGB images and the iPad depth maps, provides the best results. Interestingly, the depth maps processed with the 8× super-resolution network are capable of achieving equivalent deblurring performance to the high-resolution depth maps acquired with the Faro Focus S70 Lidar. A visualization of the 8× super-resolved depth maps against bicubic interpolation is shown in Figure 7.

#### 4.4.3. Impact of Depth Fusion and Continual Learning Adapter Design

In this study, we evaluated the design of the depth map fusion method and the continual learning strategy to create a joint deblurring model. In particular, we first assessed whether adapters are more effective than concatenating the super-resolved depth maps as an extra input channel. The results are reported in Table 6. As explained in Section 3.2.1, this is not as effective as the use of deep feature modulation and, in fact, results in a loss in the PSNR by 1.33 dB.

We then ablate the adapter design in Table 7. We first consider a variant where the convolutional attention operation at the beginning of the adapter is replaced with a sequence of convolution, LayerNorm, and PReLU. Notice how this replacement loses the analogy with the guided filter in that it uses first-order features instead of the second-order features of the convolutional attention operation and guided filter. Indeed, we can see that this design is not as effective as the one proposed in analogy to the guided filter. Moreover, we validate the use PReLU activations instead of ReLU since the naive use of ReLU activations might lead to suboptimal results due to the truncation of negative features and directional bias. Indeed, we see that ReLUs are not as effective. Finally, we assess whether having adapters at both the encoder and decoder is more effective than the proposed decoder-only solution. As mentioned in Section 3, the experiment confirms that adding encoder-side adapters is not effective and even degrades the original performance.

### 4.5. Analysis of Lidar Effectiveness

**Figure 8:**
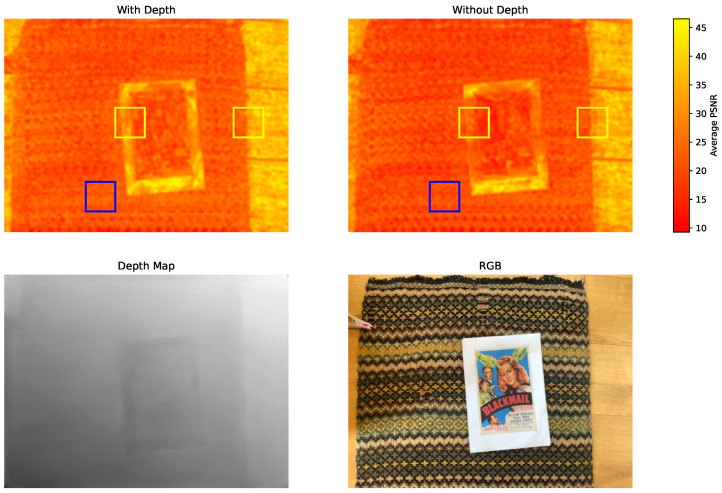
PSNR heatmap comparison between depth/non-depth network; PSNR of each pixel is averaged over 16×16 patch.

In this section, we will analyze how the depth map influences image quality and which image region benefits the most. For this purpose, Figure 8 presents a heatmap of local PSNR values, where the PSNR of each pixel is averaged over a 16×16 patch centered around it. We observe that in boundary-rich areas (highlighted by yellow bounding boxes), the depth-integrated model yields a higher PSNR. In contrast, regions with rich texture but lacking strong geometric boundaries (such as the striped carpet and the picture on the card) show little improvement. This confirms that depth information is particularly helpful in preserving sharp edges and structural transitions while offering limited gains in textured areas.

## 5. Discussion

We showed that Lidar sensors in modern smartphones can be effectively used to deblur images. In particular, we presented an efficient way of integrating Lidar depth into any state-of-the-art image deblurring model by means of lightweight neural network adapters, combined with depth super-resolution and a continual learning strategy. The achieved results are very promising and indeed show that the side information provided by mobile Lidars can significantly boost the image quality of state-of-the-art restoration neural networks. We acknowledge that there are some fundamental limitations to this sensor fusion approach. In particular, a Lidar cannot provide any information about scene textures but only 3D geometry. In the worst case of a flat textured surface, Lidar would not provide any useful data. As shown in Figure 8, when comparing PSNR heatmaps between the depth-guided and non-depth versions of our network, the difference in performance is minimal in the blanket area, which exhibits dense texture but limited geometric variation. Additionally, the range of such instruments can be limited, so scenes with objects far away from the camera may not benefit from the Lidar sensor. Finally, some geometries might be difficult to capture due to light scattering away from the camera. However, a quantitative study of this effect is currently constrained by the absence of publicly available datasets that provide paired RGB images, corresponding depth maps with varying Lidar ranges, and high-quality ground truth deblurred images. Moreover, we remark that the current study has limitations in its potential for the analysis of robustness to various Lidar sensor acquisition artifacts due to the limited availability of datasets for the Lidar-guided deblurring task and in terms of the capabilities of the smartphone acquisition platform in terms of data that are exposed.

## 6. Conclusions

We proposed using Lidar depth maps to further enhance the performance of deep deblurring models. In particular, we showed that inexpensive mobile Lidar devices can provide useful side information that improves the quality of deblurred images, especially thanks to information about object edges. The experimental results showed significant image quality improvement on synthetic and real blurred images.

While our current study demonstrates the effectiveness of depth-guided deblurring with efficient continual learning mechanisms, several challenges remain for future exploration. In particular, the focus of the current study on mobile Lidar data in iPhone smartphones where depth data is preprocessed limits the possibility of conducting an analysis on robustness to incomplete depth data or the responses of different Lidar sensors. Moreover, this study focused on scenes where the depth data could be the most useful, i.e., static, indoor scenes. Dynamic scenes could be an interesting extension but would require dedicated data. Further investigation on the performance over long-range scenes could also be of interest. Further potential avenues for future work include developing zero-shot approaches to avoid the need for extensive amounts of paired training data. Additionally, lower-complexity Lidar-guided deblurring models could be developed to enable real-time and low-memory inference on smartphones.

## Figures and Tables

**Table 1:** Hyperparameters and experimental settings. Model indicates both the original and its depth-enhanced version.

Model	Ad. Ch.	SR Ch.	Patch Sz	Loss	Epochs	Optim.	LR Policy	Init LR	Final LR
Restormer [41]	48	36	128×128	$L_{1}+L_{CD}$	200	Adam	Cosine	$5\;\times \;10^{-5}$	$1\;\times \;10^{-7}$
Stripformer [5]	64	36	128×128	$L_{1}+L_{CD}$	100	Adam	Cosine	$5\;\times \;10^{-5}$	$1\;\times \;10^{-7}$
DeblurDiNATL [62]	64	36	256×256	$L_{1}+L_{CD}$	100	Adam	Cosine	$5\;\times \;10^{-5}$	$1\;\times \;10^{-7}$
NAFNet [26]	32	36	256×256	$L_{1}+L_{CD}$	100	Adam	Cosine	$5\;\times \;10^{-5}$	$1\;\times \;10^{-7}$

**Table 2:** The effect of Lidar depth maps on state-of-the-art deblurring methods.

Model	PSNR ↑	ΔPSNR	SSIM ↑	ΔSSIM	LPIPS ↓	ΔLPIPS	Params	Runtime
Restormer [41]	34.52 dB	-	0.9318	-	0.1369	-	26.1 M	3.51 s
**Depth–Restormer**	**36.62 dB**	2.10	**0.9446**	0.0128	**0.1093**	0.0276	30.0 M	4.03 s
Stripformer [5]	35.17 dB	-	0.9337	-	0.1171	-	19.7 M	2.66 s
**Depth–Stripformer**	**36.34 dB**	1.17	**0.9412**	0.0075	**0.1118**	0.0053	22.2 M	3.12 s
DeblurDiNATL [62]	36.53 dB	-	0.9436	-	**0.1098**	0.0012	10.6 M	1.57 s
**Depth–DeblurDiNATL**	**36.72 dB**	0.19	**0.9448**	0.0012	0.1110	-	12.1M	1.90 s
NAFNet [26]	37.24 dB	-	0.9430	-	0.1160	-	17.1 M	0.32 s
**Depth–NAFNet**	**37.28 dB**	0.04	**0.9434**	0.0004	**0.1147**	0.0013	23.7 M	0.48 s

**Table 3:** Results on dataset of low-light images.

Model	Adapter	LPIPS ↓
Restormer	×	0.2418
Depth–Restormer	concat.	0.2369
Depth–Restormer	✓	**0.2332**
Stripformer	×	0.2494
Depth–Stripformer	concat.	0.2466
Depth–Stripformer	✓	**0.2435**

**Table 4:** Impact of Lidar depth maps vs. depth estimation from blurry image.

Depth Type	PSNR	ΔPSNR
None	34.52 dB	-
Estimated	35.79 dB	+1.27 dB
**Mobile Lidar**	**36.62 dB**	**+2.10 dB**

**Table 5:** Deblurring sensitivity to depth map resolution.

Depth Type	PSNR	ΔPSNR
None	34.52 dB	-
Bicubic × 8	35.78 dB	+1.26 dB
NN × 4	36.08 dB	+1.56 dB
**NN × 8**	**36.62 dB**	**+2.10 dB**
HR	36.59 dB	+2.07 dB

**Table 6:** Ablation of fusion design.

Depth	Adapter	PSNR	ΔPSNR
None	×	34.52 dB	-
NN × 8	concat.	35.24 dB	+0.72 dB
NN × 8	✓	**36.62 dB**	**+2.10 dB**

**Table 7:** Ablation of adapter design.

Adapter Design	PSNR
**Proposed**	**36.62 dB**
First-order Adapters	36.55 dB
ReLU Adapters	36.51 dB
Encoder+decoder Adapters	34.44 dB

## Data Availability

Data and code for this paper are publicly available at https://github.com/diegovalsesia/lidardeblurring (accessed on 30 October 2025).
